# Plasma Membrane Phosphatidylinositol 4-Phosphate Is Necessary for Virulence of Candida albicans

**DOI:** 10.1128/mbio.00366-22

**Published:** 2022-04-25

**Authors:** James B. Konopka

**Affiliations:** a Department of Microbiology and Immunology, Stony Brook University, Stony Brook, New York, USA

**Keywords:** *Candida albicans*, phosphatidylinositol 4-phosphate, *STT4*, fungal, hyphae, phosphatidylinositol

## Abstract

Phosphatidylinositol lipids regulate key processes, including vesicle trafficking and cell polarity. A recent study identified novel roles for phosphatidylinositol 4-phosphate (PI_4_P) in the plasma membrane of the fungal pathogen Candida albicans, including polarized hyphal growth and cell wall organization. Studies in other organisms were not able to separate the roles of PI_4_P in the plasma membrane and Golgi, but the C. albicans plasma membrane pool of PI_4_P could be selectively eliminated by deleting the *STT4* kinase, which creates PI_4_P. Interestingly, *stt4*Δ mutants were strongly defective in disseminated candidiasis in mice but were not defective in an oral infection. This suggested that abnormal exposure of β-glucan in the mutant cell walls increased recruitment of innate immune cells during disseminated infection, which is not expected to impact oral infection. These results highlight novel roles of PI_4_P and reinforce the need to test the virulence of C. albicans mutants at different host sites.

## COMMENTARY

**FIG 1:**
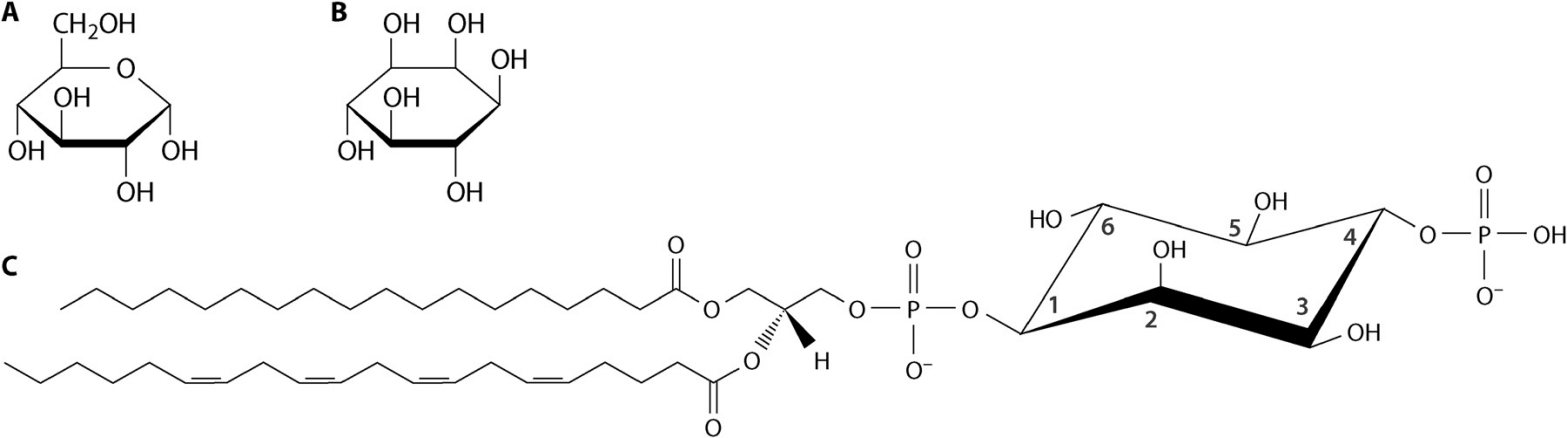
Inositol structure. Comparison of the structure of (A) glucose and (B) myo-inositol. Note that the inositol is distinct from glucose in that the ring structure is composed only of carbon-carbon bonds. (C) Structure of PI_4_P.

The sugar myo-inositol has been gaining new appreciation for its importance in regulating a wide range of functions in prokaryotic and eukaryotic cells. Inositol is a hexose, similar to glucose, but is distinct in that all six carbons are linked in a ring, whereas the glucose ring includes an oxygen (compare Fig. 1A and B). Although not as famous as the sugars that are more commonly involved in energy metabolism, such as glucose and fructose, inositol plays critical regulatory roles in the cytoplasm and in membranes, where it is used to modify lipids. Cytoplasmic inositol can be phosphorylated on all six carbon positions; some of these sites can be dually phosphorylated (pyrophosphorylated), as part of signaling mechanisms that stimulate a wide range of cellular functions (1). Inositol also forms part of the structure of membrane lipids, such as glycophosphatidylinositol modification (GPI anchor) of the C-terminal tails of cell surface proteins to anchor them in the plasma membrane, and inositol modification of ceramides to create new sphingolipid varieties. However, one of the most dynamic roles of inositol is in phosphoinositide lipids found in eukaryotic cells. Phosphatidylinositol can be phosphorylated on the 3 or 4 position of the inositol ring to create distinct phosphoinositide species (PI_3_P and PI_4_P) that can be further phosphorylated on the 5 position to create phosphatidylinositol 3,5-bisphosphate (PI_3,5_P_2_) or phosphatidylinositol 4,5-bisphosphate (PI_4,5_P_2_) (Fig. 1C). Triply phosphorylated phosphatidylinositol 3,4,5-trisphosphate occurs in mammalian cells but is not detected in fungi.

The different phosphorylated forms of phosphatidylinositol carry out distinct functions in the cell, which are not yet fully defined. Therefore, it was interesting that a recent study of the human fungal pathogen Candida albicans identified new roles for PI_4_P in the plasma membrane (2), which is the topic of this commentary. Previous studies in C. albicans and the model yeast Saccharomyces cerevisiae have shown that PI_3_P is involved in regulating intracellular vesicle trafficking, including endosomal and vacuolar membrane trafficking (3). PI_3,5_P_2_ is found on vacuolar membranes, where it plays an important role in the multivesicular body sorting pathway. PI_4_P is the major phosphoinositide species in the Golgi apparatus, where it mediates vesicular trafficking of secretory proteins from the Golgi to the plasma membrane and is required for C. albicans to undergo filamentous hyphal growth (3, 4). PI_4_P is also found at the plasma membrane (4), where it can be further phosphorylated to PI_4,5_P_2_, which is involved in regulating actin cytoskeleton organization, cell wall integrity, and heat shock response pathways (3, 5). The various forms of phosphoinositides are under dynamic regulation in the cell through the action of kinases and phosphatases that are specific for the 3′, 4′, or 5′ position (3).

A challenge in defining the roles of PI_4_P in the plasma membrane is that this phosphoinositide is also present in the Golgi, where it is involved in vesicle trafficking (4). To selectively study the role of PI_4_P at the plasma membrane, Garcia-Rodas et al. took advantage of the fact that that the pools of PI_4_P in the Golgi and plasma membrane are functionally distinct (4) and can be independently perturbed with genetic approaches (2). Although some species contain only one kinase that phosphorylates PI to generate PI_4_P, C. albicans PI_4_P is created by the Pik1 kinase in the Golgi and the Stt4 kinase at the plasma membrane. A further advantage was that the *STT4* gene could be deleted. It is not essential for growth in C. albicans, as it is in S. cerevisiae and many other organisms. This makes C. albicans an advantageous system for dissecting the plasma membrane functions of PI_4_P. As part of this study, genes were also deleted for the proteins that promote proper plasma membrane localization of Stt4, including the Efr3 membrane protein and the Ypp1 scaffold protein. Selective perturbation of the plasma membrane pool of PI_4_P was confirmed in part by using a fluorescent reporter that binds PI_4_P (GFP-PH^OSH2^-PH^OSH2^-GFP), which confirmed that there was no detectable signal at the plasma membrane in the *stt4*Δ mutant, reduced binding in the *efr3*Δ and *ypp1*Δ mutants, yet strong localization was still detected in the Golgi for all the mutant strains, as expected.

Analysis of the *stt4*Δ mutant *in vitro* revealed interesting defects that could compromise virulence, including abnormal cell wall synthesis and hyphal growth. C. albicans is multimorphic in that it can either grow as a budding yeast or switch to forming hyphae (chains of elongated cells) when placed under conditions that mimic the host environment. The mechanisms that control this morphological switch are complex, as it is under the control of a wide range of different stimuli, including nutrients, temperature, and the surrounding matrix (6). The *stt4*Δ mutant and, to a lesser degree, the *efr3*Δ and *ypp1*Δ mutants all showed defects in forming hyphae under strong inducing conditions *in vitro*. They were able to initiate short hypha-like extensions but did not maintain highly polarized filamentous growth. Identifying a role for PI_4_P in hyphal growth is significant, as this type of filamentous growth is key for biofilm formation and enhances the ability of C. albicans to grow invasively into tissues. The *stt4*Δ mutant also showed cell wall abnormalities, including increased surface exposure of β-1,3-glucan, which is expected to decrease virulence because this pathogen-associated molecular pattern is recognized by the innate immune system. There were also defects in responding to cell wall stress, including increased sensitivity to the antifungal drug caspofungin, which inhibits cell wall β-1,3-glucan synthesis.

A potential complicating factor in interpreting the effects of depleting PI_4_P at the plasma membrane is that this phosphoinositide is also needed for proper phosphatidylserine (PS) homeostasis. As shown in S. cerevisiae, oxysterol-binding proteins shuttle PI_4_P from the plasma membrane to the endoplasmic reticulum, where it is hydrolyzed by SacI, and then carry PS from the endoplasmic reticulum to the plasma membrane. This cycle depends on the gradient of PI_4_P between these membranes to drive PS transport to the plasma membrane (7). Consistent with this, a LactC2-GFP reporter indicated that there was decreased PS in the *stt4*Δ plasma membrane. However, control studies indicated that the decrease was not sufficient to account for the other phenotypes observed for PI_4_P depletion at the plasma membrane.

The roles of PI_4_P in C. albicans virulence were then assessed in two different mouse models of infection. In a model that mimics hematogenously disseminated candidiasis, the *stt4*Δ mutant was avirulent. However, a very different result occurred in a model of oropharyngeal candidiasis that mimics thrush in humans. In this case, mice infected with the *stt4*Δ mutant showed essentially the same fungal burden as mice infected with the wild-type control strain. In addition, histological analysis showed that the *stt4*Δ mutant underwent invasive hyphal growth into the tongue. Although this was surprising given the strong hyphal defect *in vitro*, other mutants with strong hyphal defects *in vitro* have been shown to grow invasively into tongue tissue (8). Thus, there must be stronger signals or different types of signal pathways activated during oral infection to promote improved hyphal growth. This raises the interesting question of how the outcomes of infection with the *stt4*Δ mutant could be so different in the oral cavity versus a disseminated infection of internal organs. The authors proposed that it is likely that the hyphal defect does not play a major role *in vivo*. Instead, they suggest that the unmasking of the β-1,3-glucan component of the cell wall may account for the stronger virulence defect in disseminated candidiasis. Exposure of this cell wall determinant is not expected to be as important during infection of the oral cavity, where one of the primary forms of immune defense is carried out by T cell activation of epithelial cells to produce antimicrobial peptides. In contrast, innate immunity plays the key role in disseminated infections. Thus, exposure of β-1,3-glucan in the *stt4*Δ mutant is expected to better recruit macrophages and neutrophils. These studies show how important it is to test the virulence potential of C. albicans mutants at different sites of infection in the host.

Like most interesting studies, the analysis of PI_4_P in C. albicans also raised some thought-provoking points. One is that it will be important to better understand why Stt4 and a plasma membrane pool of PI_4_P are not essential in C. albicans as they are in other organisms. Perhaps a clue to this is that C. albicans may have adapted different forms of regulation, since it was also observed that Stt4 and Ypp1 do not colocalize to a significant degree in the plasma membrane as they do in other organisms. Another intriguing observation was that the plasma membrane pool of PI_4,5_P_2_ appeared normal, even though PI_4,5_P_2_ is thought to be created at the plasma membrane by the phosphorylation of PI_4_P. The authors suggest that a small amount of the Golgi pool of PI_4_P may reach the membrane, where it would be quickly converted to PI_4,5_P_2_. Thus, we can look forward to more interesting results coming from the analysis of PI_4_P in C. albicans, which will serve as a good model for other organisms where it is not possible to genetically separate the Golgi and plasma membrane functions of this important phosphoinositide.
